# 5*α*,6*α*-Epoxyphytosterols and 5*α*,6*α*-Epoxycholesterol Increase Oxidative Stress in Rats on Low-Cholesterol Diet

**DOI:** 10.1155/2019/1983975

**Published:** 2019-11-15

**Authors:** Tomasz Wielkoszyński, Jolanta Zalejska-Fiolka, Joanna K. Strzelczyk, Aleksander J. Owczarek, Armand Cholewka, Aneta Krawczyk, Agata Stanek

**Affiliations:** ^1^Higher School of Strategic Planning in Dąbrowa Górnicza, Kościelna 6 St., 41-300 Dąbrowa Górnicza, Poland; ^2^Medical University of Silesia, School of Medicine with the Division of Dentistry in Zabrze, Department of Biochemistry, Jordana 19 St., 41-808 Zabrze, Poland; ^3^Medical University of Silesia, School of Medicine with the Division of Dentistry in Zabrze, Department of Medical and Molecular Biology, Jordana 19 St., 41-808 Zabrze, Poland; ^4^Medical University of Silesia, School of Pharmacy with the Division of Laboratory Medicine, Department of Statistics, Department of Instrumental Analysis, Ostrogórska 30 St, Sosnowiec 41-209, Poland; ^5^University of Silesia, Department of Medical Physics, Chelkowski Institute of Physics, Uniwersytecka 4 St., 40-007 Katowice, Poland; ^6^Medical Center and Lab, Aleja Marszałka Piłsudskiego 10, 41-300 Dąbrowa Górnicza, Poland; ^7^Medical University of Silesia, School of Medicine with the Division of Dentistry in Zabrze, Department of Internal Medicine, Angiology and Physical Medicine, Batorego 15 St., 41-902 Bytom, Poland

## Abstract

**Objective:**

Cholesterol oxidation products have an established proatherogenic and cytotoxic effect. An increased exposure to these substances may be associated with the development of atherosclerosis and cancers. Relatively little, though, is known about the effect of phytosterol oxidation products, although phytosterols are present in commonly available and industrial food products. Thus, the aim of the research was to assess the effect of 5*α*,6*α*-epoxyphytosterols, which are important phytosterol oxidation products, on redox state in rats.

**Material and Methods:**

The animals were divided into 3 groups and exposed to nutritional sterols by receiving feed containing 5*α*,6*α*-epoxyphytosterols (ES group) and 5*α*,6*α*-epoxycholesterol (Ech group) or sterol-free feed (C group). The levels of malondialdehyde (MDA), conjugated dienes (CD), and ferric reducing antioxidant potential (FRAP) were assayed in the plasma; anti-7-ketocholesterol antibodies and activity of paraoxonase-1 (PON1) were determined in serum, whereas the activity of catalase (CAT), glutathione reductase (GR), glutathione peroxidase (GPx), S-glutathione transferase (GST), and superoxide dismutase (SOD) were assayed in RBCs.

**Results:**

During the experiment, the levels of lipid peroxidation products increased, such as CD and anti-7-ketocholesterol antibodies. At the same time, the plasma levels of FRAP and serum activity of PON1 decreased alongside the reduced activity of GPx, GR, and SOD in RBCs. There was no effect of the studied compounds on the plasma MDA levels or on the activity of CAT and GST in RBCs.

**Conclusions:**

Both 5*α*,6*α*-epoxyphytosterols and 5*α*,6*α*-epoxycholesterols similarly dysregulate the *redox* state in experimental animal model and may significantly impact atherogenesis.

## 1. Introduction

Cholesterol is the most common animal sterol. It is present in every cell, as a plasma membrane component, and in the extracellular space, as a plasma lipoprotein component. Its wide bioavailability and chemical structure (monounsaturated alcohol) make cholesterol prone to oxidation, which leads to oxycholesterol formation [1]. Apart from endogenous production, oxycholesterols can also be sourced from nutrition, in particular, from cholesterol-rich foods after long-term thermal processing, gamma irradiation, or long-term storage [2].

Recently, food products containing phytosterols and phytostanols have been widely promoted. Animal experimental studies and epidemiological studies demonstrated their positive effect on lipoprotein status by, e.g., inhibiting intestinal absorption of exogenous cholesterol. Population studies show that increased intake of phytosterols and phytostanols leads to a significant decrease in total cholesterol and LDL cholesterol levels, as well as favourably affects HDL cholesterol and triacylglycerol levels [3, 4].

However, the health effects of oxidated phytosterols' intake have not been widely studied yet, although they are present in abundance in widely available and popular sterol- and stanol-containing margarines or can form during thermal processing of food products. The available literature lacks a full report on a study involving sterol administration to experimental animals which assessed sterol effect on oxidative stress. Thus, the aim of the research was to assess the effect of 5*α*,6*α*-epoxyphytosterols and 5*α*,6*α*-epoxycholesterol on oxidative stress markers in experimental animals.

## 2. Material and Methods

### 2.1. Animals

The protocol was approved by the Bioethical Committee for Animal Experimentation of the Medical University of Silesia in Katowice, Poland (approval no. 27/2007, dated April 17th, 2007). All animals received humane care in compliance with the 8th edition of the *Guide for the Care and Use of Laboratory Animals* published by the National Institute of Health [5].

Male Wistar rats, with the body weight of 130-180 g at baseline, were sourced from the Centre for Experimental Medicine, Medical University of Silesia in Katowice. During the experiment, the rats were kept on wood shaving bedding in standard single rodent cages, at the temperature of 20-25°C, with artificial lighting (a 12 h/12 h day/night cycle). The feed was administered once a day, and tap water was available *ad libitum*. Prior to the commencement of the experiment, the animals were kept in the conditions described above for an acclimation period of 2 weeks to ensure reproducible results. The rats were divided into 3 groups (15 animals each), to receive the following:

- Feed containing 5*α*,6*α*-epoxyphytosterols acetate at 100 mg per 1 kg of feed (ES group)
- Feed containing 5*α*,6*α*-epoxycholesterol acetate at 100 mg per 1 kg of feed (ECh group)
- Oxysterol-free feed (controls, C group)

Daily estimated sterol dose was 10 mg per 1 kg of animal body weight (assuming the feed intake is equal to 10% of the animal body weight). Labofeed B (Wytwórnia Pasz, Kcynia, Poland), a standard laboratory maintenance feed for rodents, was used during the study. The feed was administered for 90 days. The animals were weighted before and after the experiment. After 3 months, the rats were anaesthetised with the mixture of ketamine (50 mg/kg), droperidol (1 mg/kg), and fentanyl (0.1 mg/kg) administered i.m. and euthanised by cardiac exsanguination and cervical dislocation.

### 2.2. Synthesis of 5*α*,6*α*-Epoxycholesterol and 5*α*,6*α*-Epoxyphytosterols Acetate

The 5*α*,6*α*-epoxycholesterol acetate and 5*α*,6*α*-epoxyphytosterols acetate were synthetized, respectively, from cholesterol and sitosterol (Sigma-Aldrich, USA) by acetylation and subsequent oxidation with m-chloroperoxybenzoic acid (Sigma-Aldrich, USA) as described by McCarthy et al. [6]. Next, the oxidation mixture was purified by column chromatography on silica gel using chloroform-acetone (4:1, *v*/*v*) as a mobile phase. Fractions containing pure ester were controlled by TLC technique (silica gel plates, solvent as above), pooled, and dried under vacuum.

According to information from the manufacturer, “sitosterol” contained about 90% *β*-sitosterol and ca. 10% other phytosterols and phytostanols. Thus, its oxidation products are named as 5*α*,6*α*-epoxyphytosterols.

### 2.3. Blood Sample Collection

Blood samples were collected to tubes containing ethylenediaminetetraacetic acid (Sarstedt, S-Monovette with 1.6 mg/mL EDTA-K3) and into tubes with a clot activator (Sarstedt, S-Monovette). The blood samples were centrifuged (10 min, 900 g 4°C) and then the plasma and serum were immediately separated and stored at the temperature of –70°C, until biochemical analyses were performed. The red blood cells (RBCs) retained from the removal of EDTA plasma underwent a triple wash with cooled PBS and were lysed after the last wash in 10 mM Tris-HCl buffer pH 7.4 to obtain 10% lysates which were frozen for further analyses [7–9].

The levels of free radical damage markers, i.e., malondialdehyde (MDA), conjugated dienes (CD), and ferric reducing antioxidant power (FRAP) were assayed in EDTA plasma. Anti-7-ketocholesterol antibodies and paraoxonase-1 (PON1) activity were assayed in serum. The activity of catalase (CAT), glutathione reductase (GR), glutathione peroxidase (GPx), S-glutathione transferase (GST), and superoxide dismutase (SOD) were assayed in lysed RBCs.

### 2.4. Biochemical Analyses

#### 2.4.1. Oxidative Stress Analyses

*(1) Determination of Lipid Peroxidation Products and Antibodies against 7-Ketocholesterol*. Plasma MDA levels were determined as thiobarbituric acid reactive substances (TBARS) by spectrofluorimetric method after its derivatization with thiobarbituric acid as described by Wasowicz et al. [10] and expressed in *μ*mol/L. The inter- and intra-assay coefficients of variation (CV) were 3.5% and 5.3%, respectively.

Plasma conjugated diene (CD) levels were determined by second derivative ultraviolet spectrophotometry as described by Corongiu et al. [11] and expressed in *μ*mol/L. The inter- and intra-assay coefficients of variation (CV) were 6.2% and 8.9%, respectively.

Concentration of anti-7-ketocholesterol antibodies in serum was determined by ELISA method with the use of 7-ketocholesterol-bovine serum albumin conjugate as previously described [12]. The results were expressed as AU/mL (arbitrary units per mL). The inter- and intra-assay coefficients of variation (CV) were 8.4% and 10.2%, respectively.

*(2) Determination of Nonenzymatic Antioxidant Status*. The total antioxidant capacity of plasma was measured as the ferric reducing ability of plasma (FRAP) according to Benzie and Strain [13] and calibrated using Trolox and expressed in *μ*mol/L. The inter- and intra-assay coefficients of variation (CV) were 1.1% and 3.8%, respectively.

*(3) Determination of Activity of Antioxidant Enzymes*. Antioxidant enzyme activity was assayed in lysed RBCs obtained using 10 mM Tris-HCl buffer pH 7.2. Haemoglobin levels in lysed RBCs were estimated by Drabkin's method.

Catalase (CAT; E.C.1.11.1.6.) activity was determined in erythrocytes with the hydrogen peroxide-methanol method at 25°C developed by Johansson and Borg [14]. The method is based on the reaction of catalase with methanol in the presence of an optimal concentration of hydrogen peroxide. The obtained formaldehyde is measured spectrophotometrically at 550 nm after derivatization with Purpald as a chromogen. The enzymatic activity of catalase was expressed in kU/gHb. The inter- and intra-assay coefficients of variation (CV) were 6.8% and 9.7%, respectively.

The activity of erythrocytes glutathione reductase (GR; E.C.1.6.4.2) was determined by kinetic spectrophotometric method at 37°C using Biotech (USA) kits as per manufacturer's instructions [15, 16]. The results were expressed as International Units per a gram of haemoglobin [IU/hHb]. The inter- and intra-assay coefficients of variation (CV) were 4.2% and 6.1%, respectively.

The activity of erythrocyte glutathione peroxidase (GPx; E.C.1.11.1.9.) was determined by Paglia and Valentine's kinetic method [17] at 37°C, with t-butyl peroxide as a substrate and expressed as micromoles of NADPH oxidized per minute and normalized to one gram of haemoglobin [IU/gHb]. The inter- and intra-assay coefficients of variation (CV) were 1.8% and 3.5%, respectively.

The activity of glutathione S-transferase (GST) in RBCs was determined by kinetic spectrophotometric method [18] at 37°C using the Cayman Chemical (USA) kits. The results were expressed as International Units per a gram of haemoglobin [IU/hHb]. The inter- and intra-assay coefficients of variation (CV) were 2.7% and 3.9%, respectively.

The erythrocyte superoxide dismutase (SOD; E.C.1.15.1.1) activity was assayed using the Oyanagui method [19]. The enzymatic activity was expressed in nitrite unit (NU) in each mg of haemoglobin (Hb) [mg/Hb]. In this method, one nitrite unit (1 NU) means a 50% inhibition of nitrite ion production by SOD. The inter- and intra-assay coefficients of variation (CV) were 2.8% and 6.3%, respectively.

Paraoxonase-1 (PON-1) serum activity was assayed using the kinetic method with paraoxon (*o,o*-diethyl-*o*-(*p*-nitrophenyl)-phosphate; Sigma-Aldrich, USA) as a substrate at 37°C [20]. For cholinesterase inactivation, physostigmine salicylate (eserine) was added to serum samples ten minutes prior to the assay. One unit (1 IU) of PON-1 is the amount of enzyme sufficient to decompose 1 micromole of substrate per minute under testing conditions [IU/L]. Inter- and intra-assay coefficients (CV) of variation were 2.6% and 4.4%, respectively.

### 2.5. Statistical Analyses

Statistical analysis was performed using STATISTICA 30 PL (Tibco Inc., Palo Alto, CA, USA) and StataSE 12.0 (StataCorp LP, TX, USA) and R software (CRAN). The *p* value below 0.05 was considered statistically significant. All tests were two-tailed. Imputations were not done for missing data. Nominal and ordinal data were expressed as percentages, while interval data were expressed as mean value ± standard deviation if normally distributed or as median/interquartile range if the distribution was skewed or nonnormal. Distribution of variables was evaluated by the Shapiro-Wilk test, and homogeneity of variances was assessed using the Levene test. The comparisons were made using one-way parametric ANOVA with Tukey post hoc test.

The number of animals in each group was imposed by restrictions of the Bioethical Committee for Animal Experimentation of the Medical University of Silesia in Katowice. Nevertheless, to ensure the reliability of our results, the power analysis of the test was performed. The test power level, typically used in biomedical research, was assumed as not less than 80%.

## 3. Results

Among the markers of free radical damage, changes in their plasma concentration were only demonstrated for conjugated dienes. Their level significantly increased in the ECh group (*p* < 0.05 vs controls). Additionally, the level of anti-7-ketocholesterol antibodies increased significantly in both groups exposed to oxysterols. Whereas there were no significant differences in the levels of MDA between the study groups, there was an increasing trend demonstrated in both groups exposed to oxysterols. Plasma FRAP level was significantly lower in groups exposed to oxysterols (ES and ECh groups) as compared to controls.

In terms of antioxidant enzyme activity in RBCs, significant differences in the activity of GPx, GR, and SOD were demonstrated between the study groups, with no differences in the activity of CAT and GST. There was a significant decrease in GPx, GR, and SOD activity in RBCs demonstrated in ES and ECh groups as compared to controls, with no difference between the ES and ECh groups. The serum activity of paraoxonase-1 (PON-1) significantly decreased during the experimental exposure to oxysterols in low-cholesterol diet. The lowest PON-1 activity was demonstrated in the ECh group, with a slightly smallest reduction shown in the ES group.

**Figure 1:**
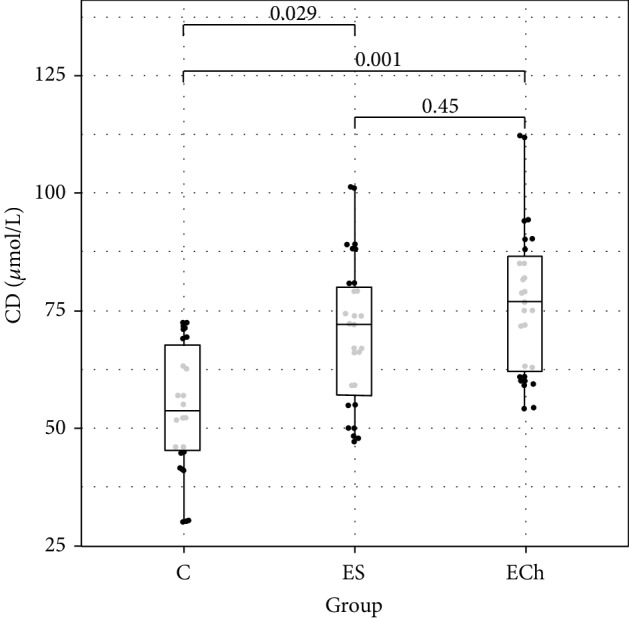
Conjugated diene (CD) levels (mean value ± standard deviation (SD)) in the plasma of rats exposed to 5*α*,6*α*-epoxycholesterol (ECh group) and 5*α*,6*α*-epoxyphytosterols (ES group) vs controls (C).

**Figure 2:**
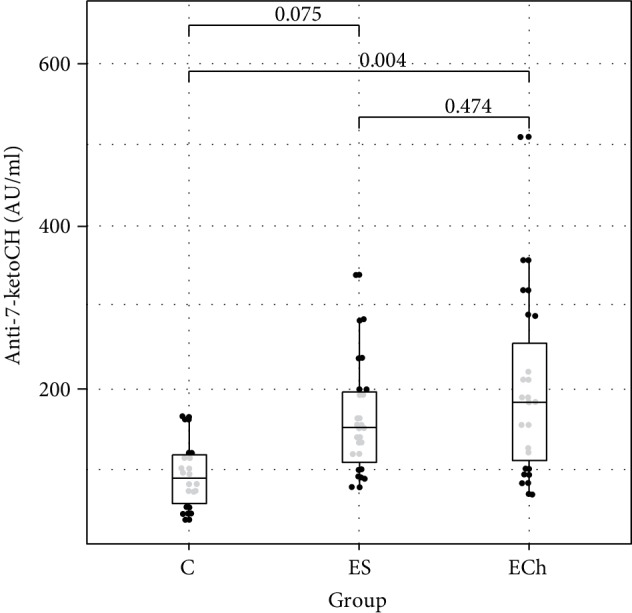
Anti-7-ketocholesterol (anti-7-ketoCH) antibody levels (mean value ± standard deviation (SD)) in the serum of rats exposed to 5*α*,6*α*-epoxycholesterol (ECh group) and 5*α*,6*α*-epoxyphytosterols (ES group) vs controls (C).

**Figure 3:**
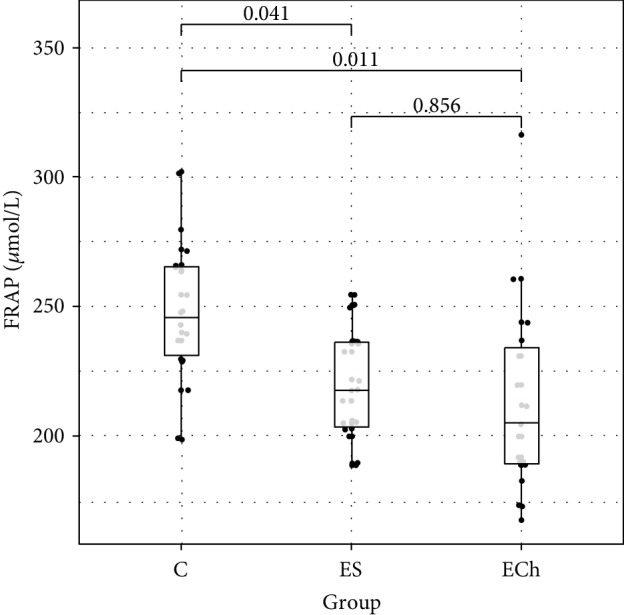
Ferric reducing antioxidant power (FRAP) levels (mean value ± standard deviation (SD)) in the plasma of rats exposed to 5*α*,6*α*-epoxycholesterol (ECh group) and 5*α*,6*α*-epoxyphytosterols (ES group) vs controls (C).

**Figure 4:**
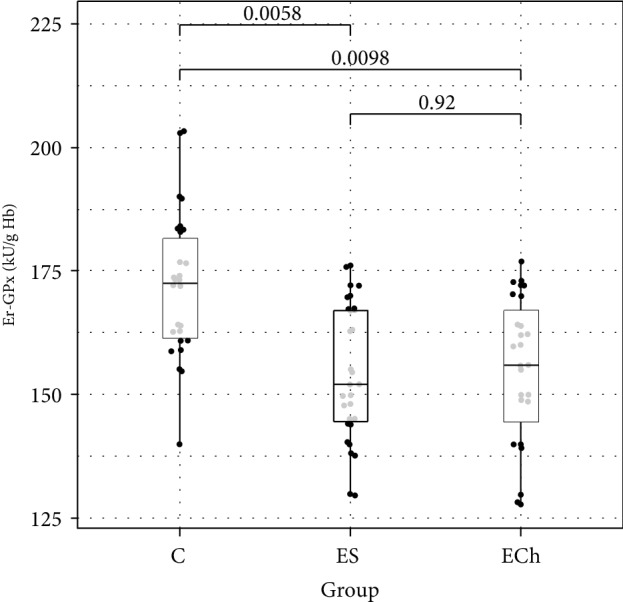
Activity of erythrocytes glutathione peroxidase (GPx) (mean value ± standard deviation (SD)) of rats exposed to 5*α*,6*α*-epoxycholesterol (ECh group) and 5*α*,6*α*-epoxyphytosterols (ES group) vs controls (C).

**Figure 5:**
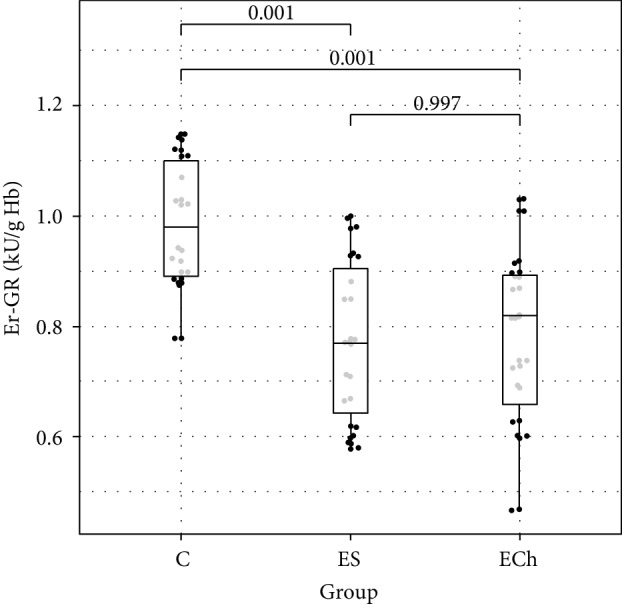
Activity of erythrocytes glutathione reductase (GR) (mean value ± standard deviation (SD)) of rats exposed to 5*α*,6*α*-epoxycholesterol (ECh group) and 5*α*,6*α*-epoxyphytosterols (ES group) vs controls (C).

**Figure 6:**
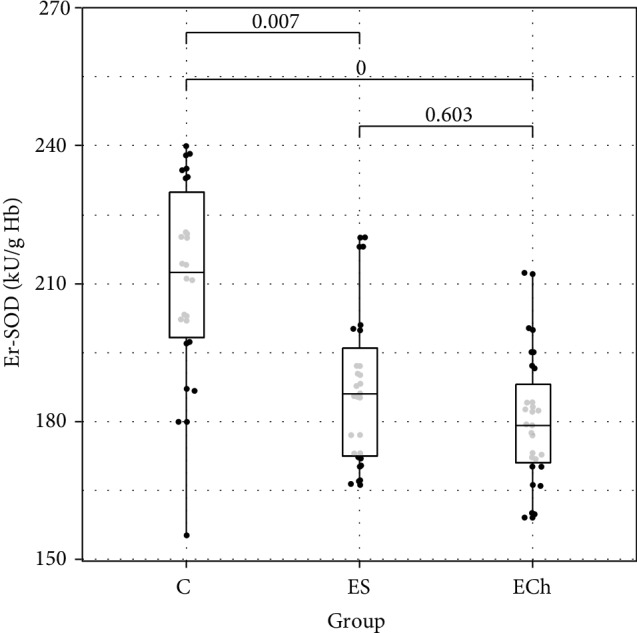
Activity of erythrocytes superoxide dismutase (SOD) (mean value ± standard deviation (SD)) of rats exposed to 5*α*,6*α*-epoxycholesterol (ECh group) and 5*α*,6*α*-epoxyphytosterols (ES group) vs controls (C).

**Figure 7:**
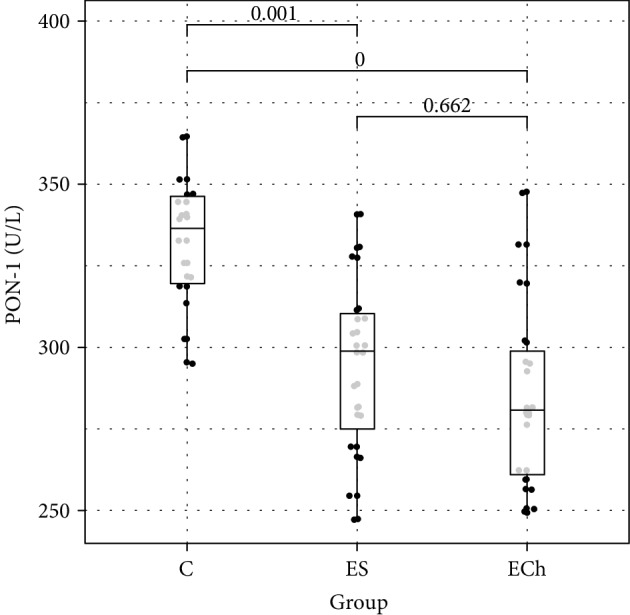
Paraoxonase-1 (PON-1) activity (mean value ± standard deviation (SD)) in the serum of rats exposed to 5*α*,6*α*-epoxycholesterol (ECh group) and 5*α*,6*α*-epoxyphytosterols (ES group) vs controls (C).

Changes to oxidative stress parameters are shown in Table 1 and Figures 1–7.

## 4. Discussion

There is ample evidence to support oxidative stress induction by oxidized cholesterol derivatives. However, there are only single reports to discuss this effect of oxyphytosterols. Until now, the only published evidence of peroxidative effect of oxyphytosterols was the study by Tomoyori et al. [21], who demonstrated an increase of plasma F2-*α* isoprostane levels in mice fed with a mixture of oxidized phytosterols, despite a simultaneous absence of their atherogenic effect. Much more is known about the harmful effect of oxidized cholesterol derivatives. Most authors agree that cytotoxic effect of oxycholesterols (such as induction of apoptosis) is primarily due to upregulated production of reactive oxygen species in cells exposed to oxycholesterols [22, 23]. The exposure of U937 cells or macrophages to 7-hydroxycholesterol led to increased apoptosis associated with the depletion of intracellular reduced glutathione [24, 25]. The exposure of U937 cells to 7-ketocholesterol or 7*α*-hydroxycholesterol also upregulated the cellular production of superoxide radical anion and downregulated nitric oxide biosynthesis [26–28], whereas the exposure to 5*α*-6*α*-epoxycholesterol did not have that effect [29]. It also seems that simultaneous exposure to the mixture of oxysterols has a stronger effect than the exposure to any individual oxysterol [22].

In our study, the concentration of conjugated dienes as early lipid peroxidation products increased significantly in rats exposed to oxysterols (both oxyphytosterols and cholesterol derivatives). It may indicate the intensified production of free radicals in animals exposed to the tested compounds. The conjugated diene assay may offer specificity at least comparable to the one of thiobarbituric acid reactive substance assay (TBARS), which is confirmed by a significantly higher concentration of conjugated dienes in plasma samples of animals exposed to 5*α*,6*α*-epoxycholesterol than in controls, with no significant differences in the concentration of MDA determined as TBARS, demonstrated in our study.

The available data show that anti-7-ketocholesterol antibody determination may be the means to indirectly monitor the severity of oxidative stress [12, 30]. The immunogenic potential of oxidized cholesterol derivatives results, for instance, from the formation of aldehyde adducts, generated during oxidation of cholesterol esters, 9-oxonanylcholesterol, and 5-oxovalerolylcholesterol, to proteins, especially apolipoprotein B [30]. It has also been shown that 7-ketocholesteryl 9-carboxinonate (oxLig-1) is a specific ligand for *α*2-glycoprotein-1. As a result, it binds specifically oxidized LDL, containing oxydized cholesterol derivatives, which is the link between autoimmune response to phospholipids (*α*2-GP-1) and atherogenesis [31]. Given that ketocholesterol is one of the major oxycholesterols, our analysis of anti-7-ketocholesterol antibody levels in a rat model provided very interesting data. We demonstrated a significant increase in the concentration of these antibodies in both groups exposed to oxysterols as compared to the controls. Since animals in any of the groups were not directly exposed to 7-ketocholesterol, the increase in the concentration of these antibodies could be solely attributable to the increase in endogenous 7-ketocholesterol formation and resultant increased immune exposure to this sterol.

The analysis of changes in the FRAP demonstrated its significant decrease in the ES and ECh groups as compared to controls. The effect of oxysterols on antioxidant activity assessed using FRAP assay, or levels of individual nonenzymatic antioxidants included in the FRAP assay, has not yet been described in any published work. Similarly, there are relatively few studies to assess the effect of oxysterols on the activity of antioxidant enzymes. Since it has been postulated that toxicity and proapoptotic effect of oxysterols are associated with an increased formation of reactive oxygen species, the majority of available papers report *in vitro* studies (mainly in cell cultures), and only a few document antioxidant enzyme changes in animals following the *in vivo* exposure to oxysterols. In rats exposed to hydrogen peroxide as an oxidative stress inductor, an increased production of oxycholesterols (25-hydroxy-, 7*α*-hydroxy- and 7-ketocholesterol), elevated MDA levels, and decreased plasma activity of CAT and SOD were observed [32]. However, it is difficult to conclude that the observed changes in enzymatic activity were directly triggered by oxidized cholesterol derivatives. Studies assessing the effect of oxycholesterols generated in a free radical-mediated process in ovarian cells showed intensified lipid peroxidation (determined as a part of TBARS) alongside increased activity of SOD and CAT [33].

In our study, the analysis of changes in the activity of superoxide dismutase in RBCs during rat exposure to 5,6-epoxysterols indicated the depletion of antioxidant defense mechanisms, which was manifested by a decrease in the activity of superoxide dismutase in ECh and ES groups. Whereas there were no significant changes in CAT activity in RBCs, the activity of GPx and GR in RBCs decreased significantly during the experimental exposure of rats to oxysterols, which was demonstrated in both ECh and ES groups in our study. We did not demonstrate significant changes to GST in RBCs.

A decrease was also demonstrated in serum paraoxonase-1 activity in groups exposed to 5*α*,6*α*-epoxycholesterol, and phytosterols 5*α*,6*α*-epoxides derivatives in this study, which may be explained by their direct effect on PON-1 biosynthesis or on oxidative stress, and may be associated with an increased formation of endogenous lipid hydroperoxides. The involvement of immune mechanisms contributing to effective elimination of PON-1 from circulation cannot be ruled out, either. Hedrick et al. demonstrated decreased serum PON-1 activity and concentration CL57BL/6 mice on atherogenic diet during the first 7 days of the experiment, whereas its respective mRNA expression in the liver remained unaffected. The finding was explained as associated with the accelerated HDL elimination from the plasma [34]. Other studies pointed to the effect of high-lipid diet on PON-1 activity. It is likely that similar mechanisms were involved, as a test meal containing thermally processed fats caused a reduction in PON-1 activity in clinically healthy volunteers, while the intake of nonoxidized fat caused an increase in the enzymatic activity of PON-1 in plasma [35].

Therefore, a reduced activity of PON-1 manifested in rodents in response to oxyphytosterols and oxycholesterols seems an important determinant of their proatherogenic profile in laboratory animals.

The limitations of the current study include a small sample size and the inability to monitor the dynamics of changes in the studied parameters, as the redox state undergoes dynamic changes throughout the exposure to the studied compounds. Similarly, it seems warranted to study the effect of other derivatives of phytosterols and cholesterol than epoxysterols in animal models. It would also be beneficial to assess the effect of those compounds on redox state in an animal model consuming atherogenic, high-cholesterol feed. What is novel about this study, though, is that it evaluates the effect of oxyphytosterols on the redox state and its associated mechanisms, as the available research mainly focuses on the effect of oxydized cholesterol derivatives on antioxidant mechanisms.

## 5. Conclusions

5*α*,6*α*-Epoxyphytosterols and 5*α*,6*α*-epoxycholesterol similarly impair the redox state in rats by increasing the production of free oxygen radicals and free radical-mediated lipid modification, as well as by affecting the mechanisms of nonenzymatic antioxidant defense and the activity of antioxidant enzymes.

## Figures and Tables

**Table 1:** Oxidative stress markers (mean value ± standard deviation (SD)) (MDA: malondialdehyde levels, anti-7-ketoCH- anti-ketocholesterol antibody levels; FRAP: total antioxidant capacity of plasma levels; CAT: activity of catalase; GPx: activity of glutathione peroxidase; GR: activity of glutathione reductase; SOD: activity of superoxide dismutase; GST: activity of glutathione S-transferase; PON-1: activity of paraoxonase-1) in plasma (p), serum (s), and erythrocytes (e) of rats fed with 5*α*,6*α*-epoxycholesterol (ECh group) and 5*α*,6*α*-epoxyphyosterols (ES group) vs controls (C).

	ECh group	ES group	C group	*p*
MDA (p) [*μ*mol/L]	3.40 ± 1.0	3.0 ± 0.55	2.7 ± 0.41	0.0545
Conjugated dienes (p) [*μ*mol/L]	76.7 ± 15.9	70.0 ± 16.2	55.0 ± 13.1	**<0.01**
Anti-7-ketoCh (s) [AU/mL]	202.2 ± 122.3	164.9 ± 75.0	92.1 ± 41.0	**<0.01**
FRAP (p) [*μ*mol/L]	214.7 ± 38.8	220.6 ± 22.1	248.8 ± 26.8	**<0.01**
CAT(e) [kIU/g Hb]	194.5 ± 18.6	187.9 ± 22.0	194.4 ± 19.3	0.600
GPx (e) [IU/g Hb]	155.0 ± 15.5	154.5 ± 14.0	171.3 ± 16.0	**<0.01**
GR (e) [IU/g Hb]	0.78 ± 0.16	0.78 ± 0.15	0.99 ± 0.12	**<0.001**
SOD (e) [NU/g Hb]	180.3 ± 14.8	187.0 ± 17.1	209.7 ± 24.5	**<0.001**
GST (e) [IU/g Hb]	0.22 ± 0.04	0.22 ± 0.03	0.24 ± 0.05	0.509
PON-1 (s) [IU/L]	286.1 ± 29.6	294.5 ± 27.8	332.5 ± 20.0	**<0.001**

## Data Availability

All data is included in the table and figures within the article.
